# Regret over the delay in childbearing decision negatively associates with life satisfaction among Japanese women and men seeking fertility treatment: a cross-sectional study

**DOI:** 10.1186/s12889-020-09025-5

**Published:** 2020-06-08

**Authors:** Tomoko Adachi, Masayuki Endo, Kazutomo Ohashi

**Affiliations:** Division of Health Science, Graduate School of Medicine, Osaka University, 1-7, Suita, Osaka, 565-0871 Japan

**Keywords:** Delay in childbearing, Regret, Life satisfaction, Informed decision-making

## Abstract

**Background:**

Currently, in developed countries, increasing numbers of women and men are delaying childbearing but begin seeking fertility treatment later in life. Some women undergoing infertility treatment develop negative feelings such as depression associated with low life satisfaction and regret over the delay in childbearing. We therefore examine the association of life satisfaction with regret over the delay in childbearing decision and infertility-related factors among Japanese women and men seeking fertility treatment.

**Methods:**

This cross-sectional study included 253 women and 196 men referred to fertility facilities in Japan from July to December 2018. Participants completed a questionnaire on life satisfaction, regret over the delay in childbearing decision, infertility-related factors and sociodemographic characteristics. Life satisfaction was measured using the Satisfaction with Life Scale (SWLS), and the degree of regret over delay in childbearing decision was measured on a 7-point Likert scale. Multiple linear regressions, conducted separately by sex, were used to analyze the association of life satisfaction with regret over the delay in childbearing decision and infertility-related factors.

**Results:**

Of the 253 women and 196 men, 102 (40.3%) women and 43 (21.9%) men answered “strongly agree” regarding their regret over the delay in childbearing decision. Among women, life satisfaction was negatively associated with regret (β = − 0.155, 95% CI [− 0.938, − 0.093], *p* = 0.017), use of assisted reproduction technology (ART) (β = − 0.135, 95% CI [− 2.977, − 0.020], *p* = 0.047). In contrast, previous live birth was positively associated with life satisfaction (β = 0.134, 95% CI [0.122, 3.739], *p* = 0.037). In men, we found no significant association of life satisfaction with regret over the delay in childbearing decision and infertility-related factors.

**Conclusions:**

Regret over the delay in childbearing decision is negatively associated with life satisfaction among Japanese women seeking fertility treatment. It may be important for women to make better informed decision regarding the timing of childbearing to not regret later in life. Health professionals should address regret over the delay in childbearing decision during fertility treatment and explore ways to spread information on fertility awareness.

## Background

Over the past decades, Japanese women and men, like those in other developed countries, have decided to delay childbearing until later in their reproductive years [1]. The proportion of first children whose mothers are over 35 years of age has increased three times over, from 9.5% in 1995 to 28.7% in 2018 [2]. However, advanced maternal and paternal age is associated with higher rates of infertility [3]; therefore, more couples face age-related infertility problems and are unable to achieve their desired number of children. A sample survey conducted in Japan every 5 years for married women under the age of 50 reported that 18.2% of couples had undergone infertility tests or treatment [4]. The increasing availability and acceptability of assisted reproductive technology (ART) – including egg freezing – allows women to delay childbearing [5–7]. Although it provides an opportunity to delay childbearing and reduces the risk of involuntary childlessness, ART does not guarantee success [8, 9].

In our preliminary study, the reasons women and men postponed childbearing were as follows: not having a partner, wanting financial security, having health problem, and being unaware of the impact of age on fertility (not published). The lack of fertility knowledge was a serious problem for Japanese individuals, although there were various reasons for delaying childbearing decision. In an international survey of 79 countries (the International Fertility Decision-making Study, IFDMS), using the Cardiff fertility knowledge scale which measures knowledge including age and fertility, scores among Japanese individuals were found to be lower than those in any other developed country [10]. As a result, delayed childbearing, likely due, at least in part, to a lack of fertility knowledge, appears to facilitate the rise of increased rates of involuntary childlessness.

Involuntarily childlessness can be a negative experience for women and men, although the degree of emotional distress can be influenced by infertility factors, such as duration of infertility and previously used ART, or parity [11–13]. Additionally, in previous qualitative studies, infertile women and men who have had delayed the timing of childbearing decision experienced regret related to this delay [14–16].

One possible psychosocial consequence of infertility is decreased life satisfaction. Life satisfaction has a cognitive judgmental aspect, and such judgement over one’s own life satisfaction is usually based on a comparison to a standard that each individual sets for her or himself [17]. Life satisfaction is the degree to which a person positively evaluates his or her life as a whole, and it represents how much the person likes that life [18]. Regarding cultural differences in life satisfaction, when retrospectively judging the level of life satisfaction, Westerners pay more attention to positive affect compared to Easterners (including Japan) [19, 20]. Additionally, Easterners rate their life satisfaction on the basis of negative experiences, while Westerners rate theirs on the basis of positive experiences [19].

MacQuillan et al. reported that life satisfaction may be significantly reduced by infertility [21]. In the other studies, life satisfaction scores of women who were unable to conceive a child after undergoing fertility treatments were significantly lower than women who could conceive [22], and women who were involuntarily childless and who resolved their fertility problem showed improved life satisfaction [23]. These findings suggest that there is an association between infertility and life satisfaction.

Given the aforementioned literature examining life satisfaction and feelings of regret in infertile women and men, an important aspect of their lives may be related to the timing of their decision on childbearing, as this may directly relate to an absence of regret over past decisions, which is an essential component of life satisfaction [24]. However, little is currently known regarding the association between life satisfaction and regret over the decision to delay childbearing decision. Thus, in the present study, we aim to examine the association between life satisfaction and regret over the decision to delay childbearing, adjusting for infertility-related factors, among Japanese women and men seeking fertility treatment. We hypothesize that regret over the delay in childbearing decision will be negatively associated with life satisfaction.

## Methods

### Study design and participants

This cross-sectional study was conducted from July to December 2018. We recruited couples seeking fertility treatment from designated fertility facilities as part of the fertility treatment support project by the Ministry of Health, Labour and Welfare, Japan. To ensure a representative sample of the general Japanese population of couples seeking for fertility treatment, we selected four facilities in East Japan and five facilities in West Japan. Most women were recruited on an individual basis and received two copies of the questionnaire: one for themselves and the other for their partners. The questionnaires were to be completed at home and returned by mail within 2 weeks.

Taking into consideration the ethics involved with conducting research using human participants, we excluded facilities that did not have a fertility counselor certified by the Japan Society for Infertility Counseling. With the intent of avoiding possible psychological burdens arising from answering our questionnaire, we asked the fertility counselors – who contacted prospective participants – to carefully explain the intentions behind this study. Fertility counselors or the first author explained the study’s intentions to patients who came to the fertility facilities, provided participants with written information about the study, and provided an informed consent form. Of the 1282 women and men recruited, 449 participants completed the survey (253 women and 196 men). Overall, the valid response rate was 35.3% for total participants; 39.5% for women and 30.6% for men.

### Measures

Life satisfaction was measured using the Satisfaction with Life Scale (SWLS). This scale was developed by Diener et al. to measure people’s cognitive judgments regarding their satisfaction with their own lives [1]. The SWLS consists of five items, as follows: (1) “In most ways, my life is close to my ideal”; (2) “The conditions of my life are excellent”; (3) “I am satisfied with my life”; (4) “So far, I have gotten the important things I want in life”; and (5) “If I could live my life over, I would change almost nothing”. Responses were provided on a scale, ranging from 1 (Strongly disagree) to 7 (Strongly agree). A total score is obtained by summing all item scores, creating a scale which can range from 5 to 35, with higher scores indicating greater life satisfaction [1]. The Japanese version of the SWLS was available as a free-of-charge download on a website provided by Diener [25].

Degree of regret over the delay in childbearing decision was measured through the question, “Would you like to have made your childbearing decision at an earlier time in your life?” Participants responded to the question using a 7-point Likert scale that ranged from strongly disagree to strongly agree. In addition, we included items that assessed sociodemographic and infertility-related characteristics - specifically, age, education, employment status and annual household income, whether participants had previously had a live birth, whether they had used ART, and the duration of their infertility.

When we first constructed the questionnaire, we conducted a preliminary test among reproductive-aged women and men. After reviewing the responses to the draft questionnaire, we repeatedly revised the questionnaire to ensure the questions were clear and easy to answer.

### Statistical analyses

First, we divided participants by sex. Data are presented as frequencies and percentages (for categorical variables) or as mean and standard deviation (for continuous variables). A difference in SWLS scores and in regret over the delay in childbearing decision were examined using an unpaired t-test and Mann-Whitney U test, respectively. Multiple linear regression analyses were conducted to examine the relationship between life satisfaction and degree of regret as well as infertility-related factors after adjusting for basic demographic factors: age, education, employment status and annual household income.

Based on the rule of thumb the multiple linear regression analyses require a sample size that is 10 times the number of independent variables, we judged our sample size to be appropriate. Data were analyzed using the IBM SPSS Statistics for Windows, Version 25.0 (IBM Corporation, Tokyo, Japan), and the level of significance was set at *p* < 0.05.

## Results

### Life satisfaction and participants’ characteristics separated by sex

Table 1 shows participants’ characteristics separated by sex. Notably, 32.8% of women used ART, and 83.1% of them were over 35 years of age. The mean score of SWLS for women was significantly lower than that for men (*M* = 21.2 vs. *M* = 22.4, *p* = 0.016).

**Table 1:** Characteristics of participants (*n* = 449)

Characteristics		Women *n* = 253	Men *n* = 196	*p* value
Age	Mean (SD)	34.9 (4.8)	36.5 (5.7)	
Duration of infertility (months)	Mean (SD)	27.3 (23.9)	25.9 (19.2)	
Previous live birth n (%)	No	214 (84.6)	167 (85.2)	
	Yes	39 (15.4)	29 (14.8)	
Used Assisted Reproduction Technology (ART) n (%)	No	170 (67.2)	129 (65.8)	
	Yes	83 (32.8)	67 (34.2)	
University Education n (%)	No	143 (56.5)	69 (35.2)	
	Yes	110 (43.5)	127 (64.8)	
Employment status n (%)	Employed full-time, Self-employed	123 (48.6)	191 (97.4)	
	Employed part-time	73 (28.9)	5 (2.6)	
	Without paid work	57 (22.5)	0 (0.0)	
Annual household income n (%)	< 6 million JPY	105 (41.5)	72 (36.7)	
	≥ 6 million JPY	148 (58.5)	124 (63.3)	
Satisfaction with Life scale	Mean (SD)	21.2 (5.2)	22.4 (5.7)	0.016 ^a^

^a^unpaired t-test

### Regret over the delay in childbearing decision separated by sex

Table 2 shows participants’ responses to the delay in childbearing decision by sex. Results indicated that 40.6% of women and 22.1% of men strongly regretted their decision. Overall, the degree of regret among women was significantly higher than that among men (*Mdn* = 6 vs. *Mdn* = 5, *p* < 0.01).

**Table 2:** Regret over choosing to delay childbearing

	Total (%)	Strongly disagree *n* (%)	Disagree *n* (%)	Somewhat disagree *n* (%)	Neither agree nor disagree *n* (%)	Somewhat agree *n* (%)	Agree *n* (%)	Strongly agree *n* (%)	Median (Interquartile range)	*p* value
Women	253 (100)	5 (2.0)	11 (4.3)	10 (4.0)	30 (11.9)	52 (20.6)	43 (17.0)	102 (40.3)	6 (5, 7)	< 0.01^a^
Men	196 (100)	7 (3.6)	10 (5.1)	19 (9.7)	30 (15.3)	33 (16.8)	54 (27.6)	43 (21.9)	5 (4, 6)	
Total	449 (100)	12 (2.7)	21 (4.7)	29 (6.5)	60 (13.4)	85 (18.9)	97 (21.6)	145 (32.3)		

^a^ Mann-Whitney U test

### Association between life satisfaction and infertility-related factors separated by sex

Table 3 shows the association between life satisfaction, regret over the delay in childbearing decision, and infertility-related factors, after adjusting for age, education level, employment status, and annual household income. A multiple linear regression analysis revealed that life satisfaction was significantly associated with regret (β = − 0.155, 95% CI [− 0.938, − 0.093], *p* = 0.017), previous live birth (β = 0.134, 95% CI [0.122, 3.739], *p* = 0.037) and use of ART (β = − 0.135, 95% CI [− 2.977, − 0.020], *p* = 0.047) in women. For men, associations between life satisfaction, regret over the delay in childbearing decision, and all infertility-related factors were non-significant.

**Table 3:** Regret and infertility-related factors associated with life satisfaction separated by sex

	Women (*n* = 253) *R* = 0.296; *R*^2^ = 0.088; Adjusted *R*^2^ = 0.058, *p* = 0.004				Men (*n* = 196) *R* = 0.267; *R*^2^ = 0.071; Adjusted *R*^2^ = 0.031, *p* = 0.081			
Characteristics	Non-standardized coefficient (B)	95% CI	Standardized coefficient (β)	*p* value	Non-standardized coefficient (B)	95% CI	Standardized coefficient (β)	*p* value
Regret	−0.515	−0.938, −0.093	−0.155	0.017	−0.401	−0.900, 0.097	−0.117	0.114
Duration of infertility	0.024	−0.004, 0.053	0.111	0.098	−0.018	−0.063, 0.027	− 0.060	0.437
Previous live birth	1.930	0.122, 3.739	0.134	0.037	2.113	−0.293, 4.519	0.132	0.085
Used assisted reproduction technology	−1.499	−2.977, −0.020	−0.135	0.047	−1.306	−3.096, 0.483	−0.109	0.152

*Note.* Adjusted by age, education level (completed university education or not), employment status (employed full-time and self-employed, or employed part-time or without paid work) and annual household income (more than 6 million JPY or not)

## Discussion

In the present study, 40.3% of women and 21.9% of men answered “strongly agree” when asked about feeling regret over the delay in childbearing decision. Notably, regret arises from the contrast between a negative outcome, owing to a perceived wrong decision and a perceived alternative, and better outcome, which may have happened if a different decision had been made [26, 27]. In a previous study, women who sought fertility treatment regretted their decision to delay childbearing, and instead, felt they should have chosen to conceive a child earlier [28]. Similarly, our participants regretted the fact that, at the time of this study, they could have already had children if they had not chosen to delay childbearing in their past.

Our results suggest that regret over the delay in childbearing decision is negatively associated with life satisfaction in women. Regret is dependent on comparison with an alternative behavior/situation that is thought to be appropriate. In previous qualitative interviews, participants’ regret over delays in finding the right partner or putting their childbearing decision on hold to achieve a successful career was due to lack of information about age-related infertility [21]. This means that they regret not choosing the appropriate behavior. This also suggests that more knowledge and information concerning the process of human fertility could have enabled them to attempt conception earlier in life, thereby reducing feelings of regret.

In the present study, previous live birth was a positive factor associated with life satisfaction among women. In a Japanese survey of men and women aged between 18 to 49 of age, researchers found that, regardless of marital status (i.e. married or unmarried), the most common reason participants had children was “Having children makes life pleasing and plentiful” [11]. Additionally, in a study in Portugal with childless women and men between 18 to 45 years of age who were randomly recruited, 99.5% of participants desired children in the future and 61.7% reported that having children would positively contribute to life satisfaction [29]. Nevertheless, a different study concluded that it cannot be said that parents are happier than nonparents, mainly because well-being is influenced by many variables that include characteristics from both parent and child [30]. Although there is no wide consensus regarding this topic, our study suggests that achieving parenthood may have a positive impact on life satisfaction for Japanese women and men seeking fertility treatment.

Additionally, we found that, for women, experience with ART was negatively related to life satisfaction, which is somewhat consistent with previous studies. For example, in previous studies, ART patients had significantly more depressive symptoms than infertile women not undergoing fertility treatments [17], and the level of depression was negatively correlated with life satisfaction [31]. In the present study, 83.1% of the women who used ART were over 35 years of age, similar to Japanese statistics in 2017, where the 35-year-old production rate per ART cycle indicated was less than 20% [32]. Ultimately, fertility treatment cannot always overcome age-related infertility and many women are experiencing psychological burdens while being treated.

In contrast to our findings with regard to women, there was no relationship observed between life satisfaction and regret over the decision to delay childbearing decision among men. Previous studies have reported that men recognize infertility as “a disconcerting event but not a tragedy” that is considered solved or accepted, whereas women see it as “a devastating experience” that affects their identity [33]. Moreover, infertile women may be more at risk of psychological distress than their partners [34].

This study has two clinical implications. First, to reduce feelings of regret related to their past decisions concerning childbearing, psychosocial counseling is recommended as an essential component of fertility treatment [35, 36], mainly because fertility treatments place psychological and physical burdens on the patient. Our results suggest that addressing the feelings of regret through counseling may have a positive impact on life satisfaction. All patients have their own personal history and characteristics, so health professionals should respect what the patients have achieved prior to planning a pregnancy and try to reduce patients’ negative affect, including feelings of regret. As fertility treatment carries no guarantee of achieving parenthood, and around 30% of the patients do not achieve it [37, 38], reducing the feeling of regret during the treatment process is also important to help patients live satisfying lives, even if they conclude treatment without having achieved parenthood.

Second, health professionals should encourage women and men to start thinking about parenthood and its timing at an earlier age. An international survey noted that the delay in childbearing decision was due to a lack of fertility knowledge – such as lack of understanding regarding age-related infertility and risk factors of infertility (e.g. smoking, weight, history of sexually transmitted infections) [10]. Although reproductive-aged Japanese individuals learn about contraception during school, they mostly learn about fertility through the mass media, followed by the internet [39]. Therefore, it is necessary to explore new ways of spreading information as widely as possible, such as creating websites and smartphone applications that incorporate the opinions of reproductive-aged women and men [40, 41]. In addition, health professionals should assess the usability of existing online information and instruct reproductive-aged women and men how to use it effectively [42]. Overall, it is important for health professionals to explore effective methods without being bound by traditional ones [41].

Previous study showed that when people made the best-informed decision possible, they could justify their decisions, even when the outcome was not one for which they had hoped [43]. Hence, to help people to live a satisfying life without major regrets later, it may be necessary to provide women and men with a better informed and satisfying reproductive life plan.

Finally, we believe that the regret owing to the delay in childbearing decision was not only a problem stemming from within the individual, but was also a social one. Health professionals should try avoiding extending medical solutions (such as ART), and the social environment should provide reproductive-aged women and men with the opportunity to start a family earlier in life without needing to sacrifice their careers, academic goals, and life expectations in order to do so [8, 13]. A reproductive life plan is provides an opportunity for reproductive-aged women and men to reflect on their interest and hopes of becoming parents and on preconception care. Just as many countries use internet to increase fertility awareness [44], using online tools and a reproductive life plan can also be helpful for Japanese reproductive-aged women and men.

This study has some limitations. First, there is an issue of causality. That is, we targeted women and men seeking fertility treatment, and given that their psychological burden of participating was considered quite heavy, we utilized a cross-sectional design. However, a cross-sectional study cannot assess causality, but only the association between lower life satisfaction and regret over delaying childbearing decision among women seeking fertility treatments. In addition, we used psychological indicators as independent and dependent variables that were unstable in their measurement. To clarify the potentially causation between life satisfaction and regret, longitudinal research with multiple indicators must be conducted, in a way that does not involve over-burdening the participants. Second, this study was limited to Japanese women and men who were seeking fertility treatment, so our findings cannot be generalized to other populations. However, we collected data from nine facilities, including seven private clinics and two units from general hospitals used for infertility treatments. Consequently, 83.1% of the women who used ART in the present study were over 35 of age and the rate was similar to Japanese ART statistics in 2017 (77.0%) [32]. Therefore, we believe that our results can be generalized to infertility patients in Japan. Third, although the overall valid response rate was low (35.3%), it was similar to a previous study of life satisfaction among infertile participants (i.e., 41%) [45]. Although the response rate tends to be low in this type of study, due to psychological burdens placed on the participant, we should further expand this research in an effort to increase the response rate. Finally, we did not investigate those who did not wish to participate. Thus, in the future, researchers should analyze participants who refuse to participate, as their burden of infertility may be so great that they refuse to participate.

## Conclusions

For women, regret over the delay in childbearing decision is negatively associated with life satisfaction. Thus, the results of our study suggest that women need to make better-informed decision concerning the timing of childbearing to reduce regret over the delay in childbearing decision later in life. It is important for health professionals to work to reduce patients’ regret over the delay in childbearing decision during fertility treatment and to explore effective methods such as the use of online tools to spread information on fertility awareness.

## Data Availability

The datasets used in this study are available from the corresponding author on reasonable request.

## Abbreviations

SWLS: Satisfaction with Life Scale
ART: Assisted Reproductive Technology
